# 8-Chloro-5-(4-phenethylpiperazin-1-­yl)pyrido[2,3-*b*][1,5]benzoxazepine

**DOI:** 10.1107/S1600536808027062

**Published:** 2008-09-06

**Authors:** Ben Capuano, Ian T. Crosby, Craig M. Forsyth, Edward J. Lloyd, Amelia Vom, Elizabeth Yuriev

**Affiliations:** a Department of Medicinal Chemistry, Victorian College of Pharmacy, Monash University (Parkville Campus), 381 Royal Park Parade, Parkville, Victoria 3052, Australia; b School of Chemistry, Monash University, Clayton, Victoria 3800, Australia

## Abstract

As part of an anti­psychotic drug discovery program, we report the crystal structure of the title compound, C_24_H_23_ClN_4_O. The mol­ecule has a tricyclic framework with a characteristic buckled V-shaped pyridobenzoxazepine unit, with the central seven-membered heterocycle in a boat configuration. The piperazine ring displays a chair conformation with the 2-phenyl-ethyl substituent assuming an equatorial orientation. There are two crystallographically independent, but virtually identical, mol­ecules in the asymmetric unit.

## Related literature

For related literature see: Andreasen *et al.* (1994 ▶, 2000 ▶); Dupont & Liégeois (2003 ▶); Petcher & Weber (1976 ▶); Capuano *et al.* (1999 ▶, 2002 ▶, 2003 ▶, 2006 ▶); Gerlach (1991 ▶); Gerson & Meltzer (1992 ▶); Liégeois *et al.* (1994 ▶, 1997 ▶, 2000 ▶); Mouithys-Mickalad *et al.* (2001 ▶); Vom (2006 ▶).
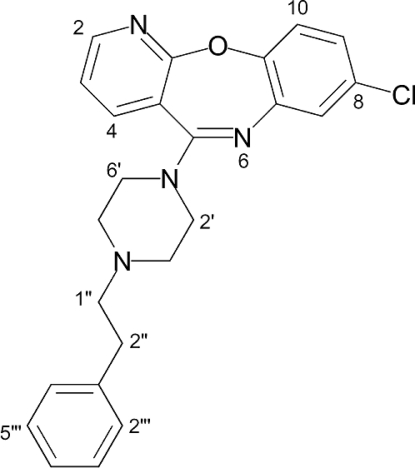


## Experimental

### 

#### Crystal data

C_24_H_23_ClN_4_O
*M* *_r_* = 418.91
Triclinic, 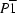

*a* = 9.9253 (2) Å
*b* = 15.0549 (3) Å
*c* = 15.9996 (3) Å
α = 107.774 (2)°
β = 95.487 (1)°
γ = 108.783 (1)°
*V* = 2104.93 (8) Å^3^
*Z* = 4
Mo *K*α radiation
μ = 0.20 mm^−1^
*T* = 123 (2) K
0.30 × 0.25 × 0.20 mm

#### Data collection

Nonius KappaCCD diffractometer
Absorption correction: none
25726 measured reflections
9532 independent reflections
6353 reflections with *I* > 2σ(*I*)
*R*_int_ = 0.084

#### Refinement

*R*[*F* ^2^ > 2σ(*F* ^2^)] = 0.055
*wR*(*F* ^2^) = 0.158
*S* = 1.00
9532 reflections
541 parameters
H-atom parameters constrained
Δρ_max_ = 0.48 e Å^−3^
Δρ_min_ = −0.32 e Å^−3^

### 

Data collection: *COLLECT* (Bruker, 2000 ▶); cell refinement: *DENZO-SMN* (Otwinowski & Minor, 1997 ▶); data reduction: *DENZO-SMN*; program(s) used to solve structure: *SHELXS97* (Sheldrick, 2008 ▶); program(s) used to refine structure: *SHELXL97* (Sheldrick, 2008 ▶); molecular graphics: *X-SEED* (Barbour, 2001 ▶); software used to prepare material for publication: *CIFTAB* (Sheldrick, 2008 ▶).

## Supplementary Material

Crystal structure: contains datablocks global, I. DOI: 10.1107/S1600536808027062/fj2144sup1.cif

Structure factors: contains datablocks I. DOI: 10.1107/S1600536808027062/fj2144Isup2.hkl

Additional supplementary materials:  crystallographic information; 3D view; checkCIF report

## Figures and Tables

**Fig. 1.**
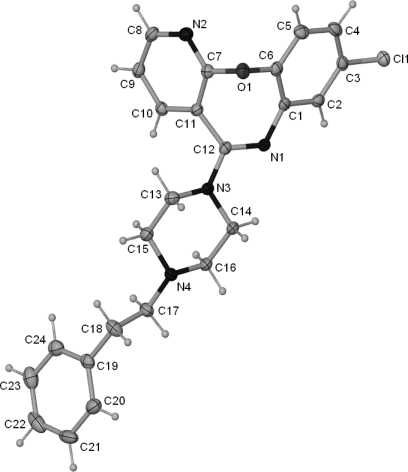
Molecular diagram of one of the crystallographically independent molecules of the title compound. Displacement ellipsoids are drawn at the 50% probability level.

## supplementary crystallographic information

### Comment

Schizophrenia  (*Gr.*,  "split mind") is a debilitating mental illness that severely impairs an individual's perception of reality, and damages a variety of emotional, behavioural and cognitive functions that we regard as specifically human (Andreasen  *et al.*, 2000). This devastating disease afflicts approximately 1% of the world population thereby making it the most common form of psychosis. The symptoms of schizophrenia are divided into two distinct classifications; positive (delusions and hallucinations) and negative (social and emotional withdrawal) (Andreasen *et al.*, 1994). Antipsychotic drugs are divided into two clinical classes; *typical*  (treat the positive symptoms and have a propensity to induce movement disorders) and *atypical* (treat both positive and negative symptoms, as well as associated cognitive deficits, and are virtually devoid of movement disorders) (Gerlach, 1991).

Clozapine is an atypical antipsychotic drug far superior in efficacy against treatment-resistant schizophrenia compared to other clinically available therapeutics. However, clozapine has been found to induce the blood disorder agranulocytosis that can, in some cases, be fatal. The synthesis component of this drug discovery programme is a continuation of previous work done in our research group (Capuano *et al.*, 2002, 2003) and is based on the structural hybridisation of two common antipsychotics, namely clozapine and haloperidol. The resulting structural series contains a tricyclic motif attached to piperazine, with an additional π-system anchored to the distal nitrogen atom of the piperazine ring system by a suitable spacer (Capuano *et al.*, 2003). The NH of the central seven-membered ring of clozapine has been isosterically replaced with oxygen, and the adjacent benzene ring replaced with a pyridine ring. This structural class, known as 'pyridobenzoxazepine', has been previously investigated by Liegeois *et al.*, with particular interest to the compound 8-chloro-5-(4-methylpiperazin-1-yl)-11*H*-pyrido[2,3-*b*] [1,5]benzoxazepine (also known as JL13), which is currently being clinically evaluated. This alternative tricyclic nucleus is predicted to enhance aqueous solubility and bioavailability compared to previously published compounds. Additionally, isosteric replacement of NH for O in structurally related compounds has been shown to significantly reduce their oxidative sensitivity towards neutrophils, and therefore lessen their hematotoxic potential (Mouithys-Mickalad *et al.*, 2001; Liegeois *et al.*, 2000; Liegeois *et al.*, 1997).

Our interest in the crystal structure of (I) was to examine the geometries of the piperazine ring and the tricyclic nucleus relative to clozapine. The crystal contains two crystallographically independent, but virtually identical molecules. The title compound (I) exhibits the characteristic buckled conformation of the pyridobenzoxazepine nucleus with the central seven-membered heterocycle in a classical boat conformation. The dihedral angle between the planes of the aromatic rings (defined as the obtuse angle subtended by the plane normals) are 112.40 (5)° (molecule 1) and 109.06 (6)° (molecule 2), which are comparable to 114° observed for JL13 (Dupont & Liégeois, 2003) and 115° observed for the prototypical atypical antipsychotic, clozapine (Petcher & Weber, 1976). The dihedral angles between the plane of the four C atoms in the piperazine ring and the chloro-substituted and pyridyl rings are 25.5 (1)° and 43.43 (6)° (molecule 1) and  26.39 (6)° and 45.42 (7)°, respectively (for clozapine, the angles are 40.5° and 31.8°, respectively (Petcher & Weber, 1976)); a consequence of the planarity of the piperazine nitrogen in the amidine moiety and the partial double bond character of N1—C12 (1.297 (3) Å and N5—C36 1.301 (2) Å. The piperazine ring adopts an almost perfect chair conformation with the phenethyl substituent assuming an equatorial orientation, by virtue of the sp^2^-like nature of the piperazine nitrogen atoms. No significant interactions between molecules of the title compound were observed.

### Experimental

The title compound (I) was synthesized (Vom, 2006) from the tricyclic lactam, 8-chlorobenzo[*b*]pyrido[3,2-*f*][1,4]oxazepin- 5(6*H*)-one (Liegeois *et al.*, 1994), and the monosubstituted piperazine, 1-phenethylpiperazine (Capuano *et al.*, 2003), in the presence of a Lewis acid, (TiCl_4_). A stirred solution of 1-phenethylpiperazine (1.00 g, 5.28 mmol) in anhydrous 1,4-dioxane (5 ml) under nitrogen was treated with a solution of titanium tetrachloride in dry toluene (1*M*, 1.1 ml, 1.1 mmol). The mixture was warmed to 50–55 °C to which a hot solution of the tricyclic lactam (250 mg, 1.01 mmol) in anhydrous dioxane (20 ml) was added. The reaction mixture was heated at reflux for 4 h after which time it was then cooled and evaporated to dryness *in vacuo*. The resulting yellow/brown residue was partitioned between sodium hydroxide solution (1*M*, 50 mL) and ethyl acetate (50 ml). The organic layer was removed and the aqueous phase was further extracted with ethyl acetate (3x50 ml). The combined organic fractions were washed with water (50 ml), dried over anhydrous sodium sulfate, and then evaporated to dryness. The resulting residue was purified by flash chromatography (ethyl acetate:methanol 97.5:2.5) and the major product evaporated to dryness. The product was recrystallized from dichloromethane/hexane as pale yellow prisms (216 mg, 51%), which were suitable for X-ray diffraction studies (mp 428 K (softens) 433 K (melts)). IR ν_max_ 1599, 1583, 1549 cm^-1. 1^H NMR (CDCl_3_) δ 2.70–2.75 (6*H*, m, H3', H5', H2''), 2.86–2.91 (2*H*, m, H1''), 3.64 (4*H*, br s, H2',H6'), 6.97 (1*H*, dd, *J* = 8.5, 2.5 Hz,H9), 7.14–7.32 (8*H*, m, H3, H7, H10, H2''', H3''', H4''', H5''', H6'''), 7.73 (1*H*, dd, *J* 7.5, 2.0 Hz, H4), 8.43 (1*H*, dd, *J* 5.0, 2.0 Hz, H2). ^13^C NMR (CDCl_3_) δ 33.0 (CH_2_), 46.9 (CH_2_), 52.7 (CH_2_), 60.1 (CH_2_), 117.6 (C_q_), 121.7 (CH), 122.3 (CH), 124.5 (CH), 126.4 (CH), 126.7 (CH), 128.6 (2 *x* CH), 131.0 (C_q_), 139.5 (C_q_), 140.7 (C_q_), 148.4 (C_q_), 151.6 (CH), 157.9 (C_q_), 164.2 (C_q_). ESI MS (20 V) m/*z* 421 (*M*[^37^Cl]H^+^, 36%), 419 (*M*[^35^Cl]H^+^, 100%). ESI high resolution MS: Found m/*z* 419.1630. Calcd. for C_24_H_23_ClN_4_O: m/*z* 419.1633.

### Refinement

All H atoms for the primary molecules were initially located in the difference Fourier map but were placed in geometrically idealized positions and constrained to ride on their parent atoms with C—H distances in the range 0.95–1.00 Å and *U*_iso_(H) = 1.2–1.5 *U*_eq_(C).

### Crystal data

**Table:**

C_24_H_23_ClN_4_O	*Z* = 4
*M**_r_* = 418.91	*F*(000) = 880
Triclinic, *P*1	*D*_x_ = 1.322 Mg m^−^^3^
Hall symbol: -P 1	Mo *K*α radiation, λ = 0.71073 Å
*a* = 9.9253 (2) Å	Cell parameters from 25726 reflections
*b* = 15.0549 (3) Å	θ = 1.5–27.5°
*c* = 15.9996 (3) Å	µ = 0.21 mm^−^^1^
α = 107.774 (2)°	*T* = 123 K
β = 95.487 (1)°	Prismatic, pale yellow
γ = 108.783 (1)°	0.30 × 0.25 × 0.20 mm
*V* = 2104.93 (8) Å^3^	

### Data collection

**Table:**

Nonius KappaCCD diffractometer	6353 reflections with *I* > 2σ(*I*)
Radiation source: fine-focus sealed tube	*R*_int_ = 0.084
graphite	θ_max_ = 27.5°, θ_min_ = 1.5°
1 deg frames in φ and ω scans	*h* = −12→12
25726 measured reflections	*k* = −19→19
9532 independent reflections	*l* = −16→20

### Refinement

**Table:**

Refinement on *F*^2^	Primary atom site location: structure-invariant direct methods
Least-squares matrix: full	Secondary atom site location: difference Fourier map
*R*[*F*^2^ > 2σ(*F*^2^)] = 0.055	Hydrogen site location: inferred from neighbouring sites
*wR*(*F*^2^) = 0.158	H-atom parameters constrained
*S* = 1.00	*w* = 1/[σ^2^(*F*_o_^2^) + (0.086*P*)^2^] where *P* = (*F*_o_^2^ + 2*F*_c_^2^)/3
9532 reflections	(Δ/σ)_max_ = 0.002
541 parameters	Δρ_max_ = 0.48 e Å^−^^3^
0 restraints	Δρ_min_ = −0.32 e Å^−^^3^

### Special details

**Table:**

Geometry. All e.s.d.'s (except the e.s.d. in the dihedral angle between two l.s. planes) are estimated using the full covariance matrix. The cell e.s.d.'s are taken into account individually in the estimation of e.s.d.'s in distances, angles and torsion angles; correlations between e.s.d.'s in cell parameters are only used when they are defined by crystal symmetry. An approximate (isotropic) treatment of cell e.s.d.'s is used for estimating e.s.d.'s involving l.s. planes.
Refinement. Refinement of *F*^2^ against ALL reflections. The weighted *R*-factor *wR* and goodness of fit *S* are based on *F*^2^, conventional *R*-factors *R* are based on *F*, with *F* set to zero for negative *F*^2^. The threshold expression of *F*^2^ > σ(*F*^2^) is used only for calculating *R*-factors(gt) *etc*. and is not relevant to the choice of reflections for refinement. *R*-factors based on *F*^2^ are statistically about twice as large as those based on *F*, and *R*- factors based on ALL data will be even larger.

### Fractional atomic coordinates and isotropic or equivalent isotropic displacement parameters (Å^2^)

**Table:**

	*x*	*y*	*z*	*U*_iso_*/*U*_eq_	
Cl1	0.43755 (6)	1.38055 (4)	1.08930 (3)	0.03010 (15)	
Cl2	0.12302 (6)	0.14811 (4)	0.23248 (3)	0.03132 (15)	
O1	0.82246 (16)	1.34506 (11)	0.83292 (9)	0.0255 (3)	
O2	−0.04349 (15)	0.18117 (10)	0.58032 (8)	0.0248 (3)	
N1	0.55743 (18)	1.17733 (13)	0.80785 (10)	0.0223 (4)	
N2	1.02695 (19)	1.32173 (13)	0.88624 (11)	0.0265 (4)	
N3	0.59265 (18)	1.05244 (13)	0.69781 (10)	0.0245 (4)	
N4	0.47574 (18)	0.86689 (13)	0.54748 (10)	0.0252 (4)	
N5	0.05941 (18)	0.35809 (13)	0.53319 (10)	0.0224 (4)	
N6	0.13408 (19)	0.17182 (13)	0.67722 (10)	0.0245 (4)	
N7	0.10986 (18)	0.48198 (12)	0.67195 (10)	0.0221 (4)	
N8	0.11770 (18)	0.67465 (12)	0.77966 (10)	0.0219 (4)	
C1	0.6055 (2)	1.26737 (16)	0.88188 (12)	0.0217 (4)	
C2	0.5157 (2)	1.27768 (16)	0.94409 (12)	0.0230 (4)	
H2	0.4327	1.2217	0.9400	0.028*	
C3	0.5486 (2)	1.36993 (16)	1.01146 (12)	0.0238 (5)	
C4	0.6674 (2)	1.45349 (16)	1.01968 (13)	0.0279 (5)	
H4	0.6862	1.5163	1.0654	0.034*	
C5	0.7592 (2)	1.44418 (16)	0.95978 (13)	0.0276 (5)	
H5	0.8423	1.5005	0.9646	0.033*	
C6	0.7283 (2)	1.35212 (16)	0.89302 (12)	0.0230 (4)	
C7	0.8879 (2)	1.28001 (16)	0.84410 (12)	0.0228 (4)	
C8	1.0898 (2)	1.25901 (17)	0.89859 (13)	0.0280 (5)	
H8	1.1890	1.2871	0.9293	0.034*	
C9	1.0194 (2)	1.15579 (17)	0.86940 (12)	0.0260 (5)	
H9	1.0689	1.1145	0.8800	0.031*	
C10	0.8740 (2)	1.11413 (16)	0.82406 (12)	0.0226 (4)	
H10	0.8224	1.0436	0.8029	0.027*	
C11	0.8060 (2)	1.17713 (15)	0.81026 (12)	0.0209 (4)	
C12	0.6466 (2)	1.13941 (15)	0.77075 (12)	0.0208 (4)	
C13	0.6737 (2)	1.03001 (16)	0.62703 (12)	0.0262 (5)	
H13A	0.7797	1.0629	0.6530	0.031*	
H13B	0.6506	1.0567	0.5804	0.031*	
C14	0.4348 (2)	1.00197 (16)	0.66368 (14)	0.0295 (5)	
H14A	0.4021	1.0285	0.6193	0.035*	
H14B	0.3838	1.0148	0.7137	0.035*	
C15	0.6332 (2)	0.91819 (16)	0.58523 (13)	0.0266 (5)	
H15A	0.6870	0.9036	0.5370	0.032*	
H15B	0.6617	0.8924	0.6313	0.032*	
C16	0.3981 (2)	0.88978 (16)	0.61953 (13)	0.0277 (5)	
H16A	0.4238	0.8630	0.6655	0.033*	
H16B	0.2919	0.8558	0.5946	0.033*	
C17	0.4348 (2)	0.75783 (16)	0.50652 (13)	0.0283 (5)	
H17A	0.3273	0.7252	0.4932	0.034*	
H17B	0.4753	0.7336	0.5502	0.034*	
C18	0.4887 (3)	0.72652 (17)	0.42041 (14)	0.0345 (6)	
H18A	0.4413	0.7450	0.3746	0.041*	
H18B	0.5951	0.7634	0.4323	0.041*	
C19	0.4567 (2)	0.61571 (17)	0.38432 (13)	0.0270 (5)	
C20	0.5436 (2)	0.57522 (18)	0.42136 (13)	0.0321 (5)	
H20	0.6227	0.6183	0.4708	0.039*	
C21	0.5179 (3)	0.47376 (19)	0.38809 (15)	0.0398 (6)	
H21	0.5809	0.4482	0.4133	0.048*	
C22	0.4013 (3)	0.40982 (19)	0.31860 (17)	0.0458 (7)	
H22	0.3828	0.3399	0.2961	0.055*	
C23	0.3108 (3)	0.4477 (2)	0.28147 (15)	0.0447 (7)	
H23	0.2294	0.4037	0.2338	0.054*	
C24	0.3391 (2)	0.55034 (19)	0.31401 (14)	0.0367 (6)	
H24	0.2773	0.5760	0.2878	0.044*	
C25	0.0493 (2)	0.26283 (15)	0.47717 (12)	0.0209 (4)	
C26	0.0878 (2)	0.25284 (16)	0.39394 (12)	0.0233 (4)	
H26	0.1293	0.3107	0.3789	0.028*	
C27	0.0653 (2)	0.15853 (16)	0.33345 (12)	0.0235 (5)	
C28	0.0012 (2)	0.07163 (16)	0.35102 (13)	0.0251 (5)	
H28	−0.0174	0.0076	0.3073	0.030*	
C29	−0.0354 (2)	0.08040 (16)	0.43400 (12)	0.0238 (5)	
H29	−0.0781	0.0221	0.4483	0.029*	
C30	−0.0094 (2)	0.17435 (15)	0.49555 (12)	0.0226 (4)	
C31	0.0867 (2)	0.22962 (16)	0.64466 (12)	0.0225 (4)	
C32	0.2615 (2)	0.21837 (17)	0.73820 (13)	0.0273 (5)	
H32	0.2987	0.1789	0.7629	0.033*	
C33	0.3407 (2)	0.32015 (16)	0.76676 (12)	0.0244 (5)	
H33	0.4299	0.3497	0.8100	0.029*	
C34	0.2875 (2)	0.37851 (16)	0.73110 (12)	0.0222 (4)	
H34	0.3399	0.4488	0.7495	0.027*	
C35	0.1563 (2)	0.33270 (15)	0.66807 (12)	0.0205 (4)	
C36	0.0998 (2)	0.38808 (15)	0.62012 (12)	0.0211 (4)	
C37	0.0757 (2)	0.50015 (15)	0.76178 (12)	0.0235 (5)	
H37A	0.1046	0.4567	0.7895	0.028*	
H37B	−0.0305	0.4830	0.7564	0.028*	
C38	0.0800 (2)	0.54901 (15)	0.62959 (12)	0.0246 (5)	
H38A	−0.0260	0.5346	0.6170	0.029*	
H38B	0.1126	0.5384	0.5720	0.029*	
C39	0.1557 (2)	0.60878 (15)	0.82070 (12)	0.0238 (5)	
H39A	0.1299	0.6206	0.8803	0.029*	
H39B	0.2621	0.6246	0.8295	0.029*	
C40	0.1610 (2)	0.65674 (15)	0.69272 (12)	0.0230 (4)	
H40A	0.2672	0.6715	0.7023	0.028*	
H40B	0.1406	0.7027	0.6649	0.028*	
C41	0.1924 (2)	0.78003 (15)	0.83789 (12)	0.0251 (5)	
H41A	0.1826	0.8231	0.8035	0.030*	
H41B	0.2974	0.7935	0.8554	0.030*	
C42	0.1336 (2)	0.80871 (16)	0.92268 (13)	0.0273 (5)	
H42A	0.0302	0.7997	0.9056	0.033*	
H42B	0.1379	0.7633	0.9556	0.033*	
C43	0.2189 (2)	0.91604 (16)	0.98384 (12)	0.0245 (5)	
C44	0.3466 (2)	0.93923 (17)	1.04455 (13)	0.0263 (5)	
H44	0.3809	0.8875	1.0460	0.032*	
C45	0.4243 (2)	1.03739 (17)	1.10308 (13)	0.0286 (5)	
H45	0.5102	1.0520	1.1449	0.034*	
C46	0.3771 (2)	1.11341 (17)	1.10059 (13)	0.0313 (5)	
H46	0.4300	1.1803	1.1407	0.038*	
C47	0.2520 (2)	1.09162 (17)	1.03929 (13)	0.0302 (5)	
H47	0.2199	1.1439	1.0366	0.036*	
C48	0.1736 (2)	0.99370 (16)	0.98195 (13)	0.0279 (5)	
H48	0.0873	0.9794	0.9406	0.033*	

### Atomic displacement parameters (Å^2^)

**Table:**

	*U*^11^	*U*^22^	*U*^33^	*U*^12^	*U*^13^	*U*^23^
Cl1	0.0300 (3)	0.0328 (3)	0.0256 (3)	0.0151 (2)	0.0060 (2)	0.0043 (2)
Cl2	0.0398 (3)	0.0298 (3)	0.0227 (3)	0.0145 (3)	0.0059 (2)	0.0056 (2)
O1	0.0273 (8)	0.0225 (8)	0.0304 (8)	0.0101 (7)	0.0088 (6)	0.0127 (6)
O2	0.0203 (7)	0.0238 (8)	0.0245 (7)	0.0041 (6)	0.0025 (5)	0.0060 (6)
N1	0.0191 (9)	0.0228 (10)	0.0223 (8)	0.0083 (8)	0.0006 (6)	0.0050 (7)
N2	0.0218 (9)	0.0254 (10)	0.0269 (9)	0.0048 (8)	0.0048 (7)	0.0062 (7)
N3	0.0176 (9)	0.0247 (10)	0.0246 (9)	0.0065 (8)	0.0023 (7)	0.0020 (7)
N4	0.0220 (9)	0.0218 (10)	0.0260 (9)	0.0075 (8)	0.0031 (7)	0.0020 (7)
N5	0.0236 (9)	0.0216 (10)	0.0213 (8)	0.0098 (8)	0.0010 (7)	0.0060 (7)
N6	0.0314 (10)	0.0224 (10)	0.0240 (9)	0.0136 (8)	0.0074 (7)	0.0100 (7)
N7	0.0289 (10)	0.0198 (9)	0.0201 (8)	0.0131 (8)	0.0030 (7)	0.0071 (7)
N8	0.0252 (9)	0.0193 (9)	0.0227 (8)	0.0101 (8)	0.0032 (7)	0.0083 (7)
C1	0.0212 (10)	0.0249 (11)	0.0203 (10)	0.0126 (9)	−0.0013 (7)	0.0073 (8)
C2	0.0211 (11)	0.0236 (11)	0.0233 (10)	0.0090 (9)	−0.0012 (8)	0.0083 (8)
C3	0.0265 (11)	0.0286 (12)	0.0199 (10)	0.0161 (10)	0.0038 (8)	0.0076 (8)
C4	0.0350 (13)	0.0218 (12)	0.0249 (11)	0.0137 (10)	−0.0007 (9)	0.0042 (8)
C5	0.0301 (12)	0.0199 (11)	0.0304 (11)	0.0074 (9)	0.0017 (9)	0.0090 (9)
C6	0.0237 (11)	0.0255 (12)	0.0234 (10)	0.0125 (9)	0.0030 (8)	0.0107 (8)
C7	0.0230 (11)	0.0232 (11)	0.0226 (10)	0.0087 (9)	0.0055 (8)	0.0086 (8)
C8	0.0207 (11)	0.0389 (14)	0.0228 (10)	0.0115 (10)	0.0030 (8)	0.0088 (9)
C9	0.0236 (11)	0.0329 (13)	0.0245 (10)	0.0143 (10)	0.0051 (8)	0.0101 (9)
C10	0.0239 (11)	0.0225 (11)	0.0213 (10)	0.0087 (9)	0.0077 (8)	0.0068 (8)
C11	0.0182 (10)	0.0226 (11)	0.0196 (9)	0.0062 (9)	0.0036 (7)	0.0061 (8)
C12	0.0187 (10)	0.0229 (11)	0.0216 (10)	0.0078 (9)	0.0025 (7)	0.0097 (8)
C13	0.0233 (11)	0.0273 (12)	0.0226 (10)	0.0048 (9)	0.0039 (8)	0.0067 (9)
C14	0.0189 (11)	0.0278 (13)	0.0330 (11)	0.0094 (9)	−0.0001 (8)	0.0000 (9)
C15	0.0211 (11)	0.0295 (13)	0.0248 (10)	0.0084 (9)	0.0046 (8)	0.0048 (9)
C16	0.0174 (10)	0.0268 (12)	0.0308 (11)	0.0070 (9)	0.0025 (8)	0.0017 (9)
C17	0.0268 (12)	0.0231 (12)	0.0284 (11)	0.0069 (10)	0.0066 (9)	0.0026 (9)
C18	0.0446 (15)	0.0251 (13)	0.0303 (12)	0.0113 (11)	0.0112 (10)	0.0061 (9)
C19	0.0288 (12)	0.0280 (12)	0.0220 (10)	0.0088 (10)	0.0110 (8)	0.0061 (9)
C20	0.0304 (13)	0.0361 (14)	0.0253 (11)	0.0086 (11)	0.0085 (9)	0.0079 (9)
C21	0.0511 (16)	0.0411 (16)	0.0432 (14)	0.0258 (13)	0.0256 (12)	0.0227 (12)
C22	0.0585 (18)	0.0264 (14)	0.0490 (15)	0.0109 (13)	0.0326 (13)	0.0077 (11)
C23	0.0328 (14)	0.0386 (16)	0.0348 (13)	−0.0027 (12)	0.0079 (10)	−0.0077 (11)
C24	0.0261 (12)	0.0428 (15)	0.0320 (12)	0.0101 (11)	0.0045 (9)	0.0044 (10)
C25	0.0196 (10)	0.0216 (11)	0.0201 (10)	0.0096 (9)	−0.0023 (7)	0.0053 (8)
C26	0.0236 (11)	0.0228 (11)	0.0232 (10)	0.0093 (9)	−0.0008 (8)	0.0088 (8)
C27	0.0254 (11)	0.0282 (12)	0.0165 (9)	0.0128 (9)	−0.0002 (8)	0.0057 (8)
C28	0.0262 (11)	0.0209 (11)	0.0237 (10)	0.0108 (9)	−0.0023 (8)	0.0021 (8)
C29	0.0189 (10)	0.0201 (11)	0.0268 (11)	0.0039 (9)	−0.0033 (8)	0.0068 (8)
C30	0.0193 (10)	0.0248 (12)	0.0208 (10)	0.0080 (9)	−0.0007 (7)	0.0057 (8)
C31	0.0227 (11)	0.0250 (12)	0.0197 (10)	0.0094 (9)	0.0061 (8)	0.0070 (8)
C32	0.0337 (12)	0.0309 (13)	0.0257 (11)	0.0193 (10)	0.0081 (9)	0.0132 (9)
C33	0.0222 (11)	0.0309 (12)	0.0226 (10)	0.0119 (9)	0.0037 (8)	0.0110 (9)
C34	0.0223 (11)	0.0237 (11)	0.0203 (10)	0.0086 (9)	0.0056 (8)	0.0073 (8)
C35	0.0211 (10)	0.0215 (11)	0.0209 (10)	0.0097 (9)	0.0057 (7)	0.0080 (8)
C36	0.0171 (10)	0.0214 (11)	0.0240 (10)	0.0067 (9)	0.0029 (7)	0.0079 (8)
C37	0.0273 (11)	0.0219 (11)	0.0251 (10)	0.0120 (9)	0.0087 (8)	0.0097 (8)
C38	0.0321 (12)	0.0222 (11)	0.0207 (10)	0.0137 (10)	0.0005 (8)	0.0072 (8)
C39	0.0276 (11)	0.0241 (12)	0.0210 (10)	0.0120 (9)	0.0048 (8)	0.0073 (8)
C40	0.0272 (11)	0.0234 (11)	0.0211 (10)	0.0117 (9)	0.0047 (8)	0.0093 (8)
C41	0.0273 (12)	0.0214 (11)	0.0257 (10)	0.0091 (9)	0.0040 (8)	0.0075 (8)
C42	0.0294 (12)	0.0228 (12)	0.0272 (11)	0.0085 (10)	0.0074 (8)	0.0062 (9)
C43	0.0273 (12)	0.0263 (12)	0.0206 (10)	0.0111 (10)	0.0078 (8)	0.0074 (8)
C44	0.0285 (12)	0.0297 (13)	0.0278 (11)	0.0144 (10)	0.0100 (8)	0.0146 (9)
C45	0.0247 (12)	0.0361 (14)	0.0237 (10)	0.0091 (10)	0.0051 (8)	0.0112 (9)
C46	0.0299 (13)	0.0275 (13)	0.0290 (11)	0.0078 (10)	0.0084 (9)	0.0026 (9)
C47	0.0346 (13)	0.0259 (12)	0.0301 (11)	0.0152 (10)	0.0064 (9)	0.0059 (9)
C48	0.0292 (12)	0.0265 (12)	0.0273 (11)	0.0122 (10)	0.0030 (8)	0.0077 (9)

### Geometric parameters (Å, °)

**Table:**

Cl1—C3	1.745 (2)	C18—H18A	0.9900
Cl2—C27	1.7442 (19)	C18—H18B	0.9900
O1—C7	1.381 (2)	C19—C20	1.388 (3)
O1—C6	1.408 (2)	C19—C24	1.389 (3)
O2—C31	1.393 (2)	C20—C21	1.381 (3)
O2—C30	1.410 (2)	C20—H20	0.9500
N1—C12	1.297 (3)	C21—C22	1.373 (4)
N1—C1	1.401 (2)	C21—H21	0.9500
N2—C7	1.326 (3)	C22—C23	1.386 (4)
N2—C8	1.338 (3)	C22—H22	0.9500
N3—C12	1.363 (2)	C23—C24	1.392 (3)
N3—C14	1.466 (3)	C23—H23	0.9500
N3—C13	1.469 (2)	C24—H24	0.9500
N4—C16	1.463 (2)	C25—C26	1.398 (3)
N4—C17	1.467 (3)	C25—C30	1.405 (3)
N4—C15	1.470 (3)	C26—C27	1.385 (3)
N5—C36	1.301 (2)	C26—H26	0.9500
N5—C25	1.406 (2)	C27—C28	1.385 (3)
N6—C31	1.320 (3)	C28—C29	1.389 (3)
N6—C32	1.348 (3)	C28—H28	0.9500
N7—C36	1.369 (3)	C29—C30	1.379 (3)
N7—C38	1.462 (3)	C29—H29	0.9500
N7—C37	1.472 (2)	C31—C35	1.390 (3)
N8—C41	1.464 (2)	C32—C33	1.380 (3)
N8—C39	1.466 (3)	C32—H32	0.9500
N8—C40	1.467 (2)	C33—C34	1.387 (3)
C1—C6	1.402 (3)	C33—H33	0.9500
C1—C2	1.405 (3)	C34—C35	1.391 (3)
C2—C3	1.387 (3)	C34—H34	0.9500
C2—H2	0.9500	C35—C36	1.486 (3)
C3—C4	1.380 (3)	C37—C39	1.508 (3)
C4—C5	1.391 (3)	C37—H37A	0.9900
C4—H4	0.9500	C37—H37B	0.9900
C5—C6	1.384 (3)	C38—C40	1.517 (3)
C5—H5	0.9500	C38—H38A	0.9900
C7—C11	1.395 (3)	C38—H38B	0.9900
C8—C9	1.384 (3)	C39—H39A	0.9900
C8—H8	0.9500	C39—H39B	0.9900
C9—C10	1.393 (3)	C40—H40A	0.9900
C9—H9	0.9500	C40—H40B	0.9900
C10—C11	1.384 (3)	C41—C42	1.524 (3)
C10—H10	0.9500	C41—H41A	0.9900
C11—C12	1.495 (3)	C41—H41B	0.9900
C13—C15	1.506 (3)	C42—C43	1.510 (3)
C13—H13A	0.9900	C42—H42A	0.9900
C13—H13B	0.9900	C42—H42B	0.9900
C14—C16	1.520 (3)	C43—C48	1.388 (3)
C14—H14A	0.9900	C43—C44	1.394 (3)
C14—H14B	0.9900	C44—C45	1.392 (3)
C15—H15A	0.9900	C44—H44	0.9500
C15—H15B	0.9900	C45—C46	1.379 (3)
C16—H16A	0.9900	C45—H45	0.9500
C16—H16B	0.9900	C46—C47	1.386 (3)
C17—C18	1.521 (3)	C46—H46	0.9500
C17—H17A	0.9900	C47—C48	1.385 (3)
C17—H17B	0.9900	C47—H47	0.9500
C18—C19	1.501 (3)	C48—H48	0.9500
			
C7—O1—C6	108.51 (15)	C21—C22—C23	119.7 (2)
C31—O2—C30	107.85 (14)	C21—C22—H22	120.1
C12—N1—C1	122.45 (17)	C23—C22—H22	120.1
C7—N2—C8	116.26 (19)	C22—C23—C24	120.0 (2)
C12—N3—C14	119.65 (17)	C22—C23—H23	120.0
C12—N3—C13	122.36 (17)	C24—C23—H23	120.0
C14—N3—C13	111.97 (15)	C19—C24—C23	120.8 (2)
C16—N4—C17	109.81 (16)	C19—C24—H24	119.6
C16—N4—C15	108.62 (15)	C23—C24—H24	119.6
C17—N4—C15	111.58 (17)	C26—C25—C30	117.01 (18)
C36—N5—C25	121.46 (18)	C26—C25—N5	118.53 (18)
C31—N6—C32	116.07 (18)	C30—C25—N5	124.21 (17)
C36—N7—C38	120.12 (15)	C27—C26—C25	119.9 (2)
C36—N7—C37	120.21 (17)	C27—C26—H26	120.0
C38—N7—C37	112.65 (16)	C25—C26—H26	120.0
C41—N8—C39	110.58 (15)	C26—C27—C28	122.30 (19)
C41—N8—C40	110.43 (16)	C26—C27—Cl2	119.27 (17)
C39—N8—C40	107.81 (16)	C28—C27—Cl2	118.42 (15)
N1—C1—C6	124.50 (18)	C27—C28—C29	118.42 (18)
N1—C1—C2	117.77 (18)	C27—C28—H28	120.8
C6—C1—C2	117.31 (18)	C29—C28—H28	120.8
C3—C2—C1	119.8 (2)	C30—C29—C28	119.5 (2)
C3—C2—H2	120.1	C30—C29—H29	120.3
C1—C2—H2	120.1	C28—C29—H29	120.3
C4—C3—C2	122.16 (19)	C29—C30—C25	122.73 (18)
C4—C3—Cl1	118.81 (15)	C29—C30—O2	118.25 (18)
C2—C3—Cl1	119.03 (17)	C25—C30—O2	119.02 (17)
C3—C4—C5	118.86 (19)	N6—C31—C35	125.67 (19)
C3—C4—H4	120.6	N6—C31—O2	116.04 (18)
C5—C4—H4	120.6	C35—C31—O2	118.28 (18)
C6—C5—C4	119.4 (2)	N6—C32—C33	123.6 (2)
C6—C5—H5	120.3	N6—C32—H32	118.2
C4—C5—H5	120.3	C33—C32—H32	118.2
C5—C6—C1	122.44 (19)	C32—C33—C34	118.69 (19)
C5—C6—O1	118.19 (19)	C32—C33—H33	120.7
C1—C6—O1	119.34 (17)	C34—C33—H33	120.7
N2—C7—O1	116.16 (19)	C33—C34—C35	119.05 (19)
N2—C7—C11	125.0 (2)	C33—C34—H34	120.5
O1—C7—C11	118.86 (18)	C35—C34—H34	120.5
N2—C8—C9	124.1 (2)	C31—C35—C34	116.89 (19)
N2—C8—H8	117.9	C31—C35—C36	120.65 (17)
C9—C8—H8	117.9	C34—C35—C36	122.14 (19)
C8—C9—C10	118.2 (2)	N5—C36—N7	119.44 (19)
C8—C9—H9	120.9	N5—C36—C35	124.18 (18)
C10—C9—H9	120.9	N7—C36—C35	115.85 (16)
C11—C10—C9	119.0 (2)	N7—C37—C39	110.22 (16)
C11—C10—H10	120.5	N7—C37—H37A	109.6
C9—C10—H10	120.5	C39—C37—H37A	109.6
C10—C11—C7	117.41 (18)	N7—C37—H37B	109.6
C10—C11—C12	122.08 (19)	C39—C37—H37B	109.6
C7—C11—C12	120.12 (19)	H37A—C37—H37B	108.1
N1—C12—N3	119.41 (18)	N7—C38—C40	108.73 (15)
N1—C12—C11	123.82 (17)	N7—C38—H38A	109.9
N3—C12—C11	116.22 (17)	C40—C38—H38A	109.9
N3—C13—C15	109.85 (17)	N7—C38—H38B	109.9
N3—C13—H13A	109.7	C40—C38—H38B	109.9
C15—C13—H13A	109.7	H38A—C38—H38B	108.3
N3—C13—H13B	109.7	N8—C39—C37	110.54 (16)
C15—C13—H13B	109.7	N8—C39—H39A	109.5
H13A—C13—H13B	108.2	C37—C39—H39A	109.5
N3—C14—C16	109.06 (17)	N8—C39—H39B	109.5
N3—C14—H14A	109.9	C37—C39—H39B	109.5
C16—C14—H14A	109.9	H39A—C39—H39B	108.1
N3—C14—H14B	109.9	N8—C40—C38	111.45 (16)
C16—C14—H14B	109.9	N8—C40—H40A	109.3
H14A—C14—H14B	108.3	C38—C40—H40A	109.3
N4—C15—C13	111.03 (18)	N8—C40—H40B	109.3
N4—C15—H15A	109.4	C38—C40—H40B	109.3
C13—C15—H15A	109.4	H40A—C40—H40B	108.0
N4—C15—H15B	109.4	N8—C41—C42	113.44 (17)
C13—C15—H15B	109.4	N8—C41—H41A	108.9
H15A—C15—H15B	108.0	C42—C41—H41A	108.9
N4—C16—C14	112.14 (18)	N8—C41—H41B	108.9
N4—C16—H16A	109.2	C42—C41—H41B	108.9
C14—C16—H16A	109.2	H41A—C41—H41B	107.7
N4—C16—H16B	109.2	C43—C42—C41	112.03 (17)
C14—C16—H16B	109.2	C43—C42—H42A	109.2
H16A—C16—H16B	107.9	C41—C42—H42A	109.2
N4—C17—C18	113.11 (18)	C43—C42—H42B	109.2
N4—C17—H17A	109.0	C41—C42—H42B	109.2
C18—C17—H17A	109.0	H42A—C42—H42B	107.9
N4—C17—H17B	109.0	C48—C43—C44	118.2 (2)
C18—C17—H17B	109.0	C48—C43—C42	121.78 (19)
H17A—C17—H17B	107.8	C44—C43—C42	120.0 (2)
C19—C18—C17	111.98 (18)	C45—C44—C43	120.7 (2)
C19—C18—H18A	109.2	C45—C44—H44	119.7
C17—C18—H18A	109.2	C43—C44—H44	119.7
C19—C18—H18B	109.2	C46—C45—C44	120.2 (2)
C17—C18—H18B	109.2	C46—C45—H45	119.9
H18A—C18—H18B	107.9	C44—C45—H45	119.9
C20—C19—C24	117.9 (2)	C45—C46—C47	119.6 (2)
C20—C19—C18	120.6 (2)	C45—C46—H46	120.2
C24—C19—C18	121.5 (2)	C47—C46—H46	120.2
C21—C20—C19	121.6 (2)	C48—C47—C46	120.1 (2)
C21—C20—H20	119.2	C48—C47—H47	119.9
C19—C20—H20	119.2	C46—C47—H47	119.9
C22—C21—C20	120.0 (3)	C47—C48—C43	121.1 (2)
C22—C21—H21	120.0	C47—C48—H48	119.4
C20—C21—H21	120.0	C43—C48—H48	119.4
